# Analysis of Catalase-Induced Activation of Intracellular Cell Signaling in Macrophages

**DOI:** 10.3390/antiox15030366

**Published:** 2026-03-13

**Authors:** Kaiwen Mu, Ningjian Liang, Maidinai Sabier, Yu-Hsuan Liao, David. D. Kitts

**Affiliations:** 1 Food Nutrition and Health, Faculty of Land and Food Systems, The University of British Columbia, Vancouver, BC V6T-1Z4, Canada; kaiwen.panda@gmail.com (K.M.); msabier@student.ubc.ca (M.S.); jessicaliao326@gmail.com (Y.-H.L.); 2 Department of Animal, Veterinary & Food Science, College of Agricultural and Life Sciences, Moscow, ID 83844, USA; nliang@uidaho.edu

**Keywords:** redox status, extracellular redox homeostasis, cell signaling, macrophages

## Abstract

Hydrogen peroxide (H_2_O_2_) is a key extracellular redox signaling molecule that regulates diverse physiological processes, including immune cell activation and proliferation. However, its role in maintaining extracellular redox balance and mediating intercellular signaling remains underexplored. In this study, we investigated how extracellular depletion of H_2_O_2_ by catalase modulates intracellular signaling pathways in macrophages. Catalase treatment effectively depleted extracellular H_2_O_2_ in a concentration- and time-dependent manner, leading to activation of mitogen-activated protein kinase (MAPK) pathways, including extracellular signal-regulated kinase (ERK), c-Jun N-terminal kinase (JNK), and p38, as well as nuclear translocation of the nuclear factor κB (NF-κB) p65 subunit. Perturbation of extracellular redox status resulted in robust upregulation of inflammatory and oxidative stress–related genes, including cyclooxygenase-2 (COX-2), C-C motif chemokine ligand 5 (CCL5), inducible nitric oxide synthase (iNOS), and nicotinamide adenine dinucleotide phosphate (NADPH) oxidase. This transcriptional response was accompanied by increased nitric oxide (NO) production and enhanced nuclear translocation and DNA-binding activity of nuclear factor erythroid 2–related factor 2 (Nrf2). Mechanistically, our data suggest that NO-mediated S-nitrosylation contributes to activation of the cellular antioxidant response. In addition, catalase-mediated depletion of extracellular H_2_O_2_ significantly (*p* < 0.05) suppressed 5-bromo-2′-deoxyuridine (BrdU) incorporation, indicating inhibition of macrophage proliferation. Together, these findings demonstrate that extracellular H_2_O_2_ functions as a physiological redox signal that maintains cellular homeostasis, and that its removal triggers a coordinated intracellular response involving both inflammatory activation and antioxidant defense. This study highlights the critical role of extracellular redox balance in shaping macrophage function and provides mechanistic insight into how changes in the oxidative environment regulate downstream immune signaling pathways.

## 1. Introduction

Hydrogen peroxide (H_2_O_2_) is a key redox-active molecule generated as a byproduct of normal cellular metabolism and plays a dual role as both a metabolic intermediate and a signaling mediator [1]. Catalase, a multi-subunit, heme-containing oxidoreductase enzyme, is important for rapidly catalyzing the decomposition of H_2_O_2_ into harmless water (H_2_O) and molecular oxygen (O_2_), thereby preventing the conversion of H_2_O_2_ into highly reactive hydroxyl radicals that trigger oxidative stress. In recent years, H_2_O_2_ has emerged as a crucial second messenger in redox signaling, modulating essential cellular processes such as proliferation, differentiation, apoptosis, and immune responses [1]. While intracellular hydrogen peroxide is tightly regulated by antioxidant systems, extracellular H_2_O_2_ also plays critical signaling roles, particularly in mediating intercellular communication and influencing immune cell behavior [2,3]. At physiological concentrations, extracellular H_2_O_2_ facilitates wound healing and immune defense, whereas aberrant accumulation or depletion of H_2_O_2_ has been implicated in various pathological conditions, including cancer, cardiovascular diseases, and chronic inflammation [4,5,6,7].

Maintaining extracellular redox balance is essential for cellular homeostasis. Excess H_2_O_2_ contributes to oxidative stress and tissue damage, whereas insufficient levels can impair protective redox signaling [8]. Extracellular redox status influences intracellular signal transduction by modulating redox-sensitive transcription factors and signaling pathways, such as nuclear factor kappa-light-chain-enhancer of activated B cells (NF-κB) and nuclear factor erythroid 2–related factor 2 (Nrf2) [9]. Numerous studies have shown that shifts in extracellular redox potential are associated with pathophysiological processes, including aging, diabetes, neurodegeneration, and cardiovascular disease [10,11,12]. On this basis, changes in extracellular redox markers have also been proposed as potential diagnostic indicators of disease onset or progression [10,11,12].

Macrophages are highly sensitive to redox changes and function as critical regulators of host defense, inflammation, and immune surveillance [13]. These innate immune cells exhibit remarkable functional plasticity, adapting to physiological or pathological cues through phenotype switching and reprogramming of signaling networks [14]. Macrophage function is closely linked to the redox microenvironment, which influences inflammatory status, phagocytic capacity, and cytokine production [15]. Chronic redox imbalance can compromise their reparative and homeostatic roles, thereby contributing to disease pathogenesis [15].

Given the emerging interest in redox modulation as a therapeutic strategy, it is important to understand how extracellular redox changes influence macrophage behavior. In particular, H_2_O_2_ serves as a key component of the extracellular redox environment. The enzymatic degradation of extracellular H_2_O_2_ provides a useful tool to investigate how perturbations in redox balance affect downstream cellular responses [16].

In this study, we employed catalase, an oxidoreductase enzyme that decomposes H_2_O_2_ into water and oxygen, to selectively regulate extracellular H_2_O_2_ concentrations and examine their effects on redox signaling in RAW 264.7 macrophages. By introducing catalase at concentrations ranging from low to relatively high and monitoring the degradation of H_2_O_2_ over time, we aimed to characterize the temporal dynamics of extracellular H_2_O_2_ clearance. This approach allowed us to subsequently assess the concentration- and time-dependent effects of catalase treatment on signaling pathways and redox balance in RAW 264.7 macrophages.

Subsequent experiments investigated the intracellular consequences of extracellular H_2_O_2_ depletion, with a particular focus on redox-sensitive inflammatory signaling pathways. We assessed nitric oxide (NO) production as a regulator of macrophage activation and evaluated the activation of NF-κB and the expression of Nrf2, two key transcription factors involved in inflammation and antioxidant defense. By integrating extracellular redox modulation with intracellular readouts, this study aims to elucidate how changes in extracellular H_2_O_2_ influence macrophage signaling and contribute to a broader understanding of redox biology in health and disease.

## 2. Materials and Methods

### 2.1. Evaluation of Catalase Degradation Capacity

Catalase (EC 1.11.1.6) was purchased from Cayman Chemical Company (Ann Arbor, MI, USA). The degradation capacity of catalase was evaluated using the H_2_O_2_ Cell-Based Assay Kit (Item No. 600050) (Cayman Chemical, Ann Arbor, MI, USA). Fluorescence was measured using an excitation wavelength of 530–560 nm and an emission wavelength of 590 nm, corresponding to the peak fluorescence of resorufin (Cayman Chemical, Ann Arbor, MI, USA). The H_2_O_2_ standard curve was prepared according to the kit instructions (Cayman Chemical, Ann Arbor, MI, USA). Each time point was measured in triplicate to ensure reproducibility and minimize experimental variability.

### 2.2. Cell Culture

RAW 264.7 (TIB-71) cells were purchased from the American Type Culture Collection (ATCC, Manassas, VA, USA). Cells were cultured in Dulbecco’s Modified Eagle’s Medium (DMEM) supplemented with 10% fetal bovine serum (FBS; Invitrogen, Carlsbad, CA, USA), 100 U/mL penicillin (Sigma, St. Louis, MO, USA), and 100 µg/mL streptomycin. Cells were maintained at 37 °C in a humidified atmosphere containing 5% CO_2_, and the culture medium was replaced every 2–3 days. Cells between passages 31 and 35 were used for all experiments.

### 2.3. Mitogen-Activated Protein Kinase (MAPK)

RAW 264.7 cells (TIB-71) were seeded at a density of 1 × 10^5^ cells per well in a 96-well plate and cultured overnight, followed by treatment with catalase for 1 h. Cells were then collected using M-PER™ Mammalian Protein Extraction Reagent (Cat. No. 78501, Thermo Fisher Scientific, Waltham, MA, USA) according to the manufacturer’s instructions. The JNK 1/2 (pT183/Y185 + Total) ELISA Kit (ab176662), p38 MAPK alpha (pT180/Y182 + Total) ELISA Kit (ab221013), and ERK 1/2 (pT202/Y204 + Total) ELISA Kit (ab176660) were purchased from Abcam (Toronto, ON, Canada).

### 2.4. Analysis of p65 Binding Activity by Transactivation Assay

RAW 264.7 cells (TIB-71) were seeded at a density of 3 × 10^6^ cells per well in a 6-well plate and cultured overnight, followed by treatment with catalase for 1.5 h. Subsequently, nuclear proteins were extracted using a Nuclear Extraction Kit (Cayman Chemical, Item No. 10009277). NF-κB (p65) activity was then measured using an NF-κB (p65) transcription factor ELISA assay kit (Item No. 10007889, Cayman Chemical Company, Ann Arbor, MI, USA).

### 2.5. Real-Time PCR Microarrays

RAW 264.7 cells (TIB-71) were seeded at a density of 3 × 10^6^ cells per well in a 6-well plate and cultured overnight, followed by treatment with catalase for four hours. Total RNA was isolated from RAW 264.7 cells using the RNeasy Mini Kit (Qiagen, Valencia, CA, USA), followed by reverse transcription using the RT^2^ First Strand Kit (Qiagen, Valencia, CA, USA). Subsequently, RNA was converted to complementary DNA (cDNA) using the RT^2^ Reaction Ready First Strand Synthesis Kit (Qiagen, Valencia, CA, USA). The resulting cDNA was analyzed using the RT^2^ Profiler™ PCR Array (Qiagen, Valencia, CA, USA). Details of the array are provided (Table S1).

PCR amplification was performed according to the manufacturer’s instructions using a CFX96 Touch Real-Time PCR Detection System (Bio-Rad Laboratories, Hercules, CA, USA), and cDNA was amplified using RT^2^ SYBR Green qPCR Mastermix (Qiagen, Valencia, CA, USA). The PCR thermal cycling conditions were as follows: 95 °C for 10 min, followed by 40 cycles of 95 °C for 15 s and 60 °C for 60 s. A melting curve analysis from 60 °C to 95 °C was performed to confirm amplification specificity.

Gene expression data were normalized to the housekeeping gene β-actin and analyzed using the 2^(−ΔΔCt)^ method. The ΔΔCt value was calculated as:

ΔΔCt = (Ct_treatment,target − Ct_treatment,reference) − (Ct_control,target − Ct_control,reference)

A minimum two-fold increase or decrease in gene expression compared with the control was considered biologically significant. Each experiment consisted of three independent biological replicates. Statistical significance of fold changes was determined using a paired Student’s *t*-test, and results were considered significant at *p* < 0.05.

### 2.6. Analysis of Nrf-2 Binding Activity by Transactivation Assay

RAW 264.7 cells (TIB-71) were seeded at a density of 3 × 10^6^ cells per well in 6-well plates and cultured overnight, followed by treatment with catalase for 24 h. Subsequently, nuclear proteins were extracted using a nuclear extraction kit, and Nrf2 binding activity was measured using an Nrf2 transcription factor ELISA assay kit (Item No. 600590, Cayman Chemical Company, Ann Arbor, MI, USA).

### 2.7. Nitric Oxide (NO) Measurement

RAW 264.7 cells (TIB-71) were seeded at a density of 1 × 10^5^ cells per well in a 96-well plate and cultured overnight, followed by treatment with catalase for 20 h. Nitric oxide production in the cell culture supernatant was measured using the Griess assay. The color change was measured using a spectrophotometer (Multiskan Ascent, ThermoLabsystems, Helsinki, Finland) at 540 nm.

### 2.8. BrdU Cell Proliferation Assay

RAW 264.7 cells (TIB-71) were seeded at a density of 1 × 10^5^ cells per well in a 96-well plate and cultured overnight, followed by treatment with catalase for 24 h. BrdU incorporation into newly synthesized DNA of actively proliferating cells was measured using a BrdU Cell Proliferation ELISA kit according to the manufacturer’s instructions (ab126556, Abcam, Toronto, ON, Canada).

### 2.9. Statistical Analysis

Data was analyzed by a one-way ANOVA using MINITAB 19 software (Minitab Inc., State College, PA, USA). Significant differences were compared using Tukey’s test with *p* < 0.05 representing a statistically significant difference. Key comparisons were independently confirmed using a non-parametric Kruskal–Wallis test.

## 3. Results

### 3.1. Extracellular H_2_O_2_ Depletion by Catalase: Dose- and Time-Dependent Kinetics

**Figure 1:**
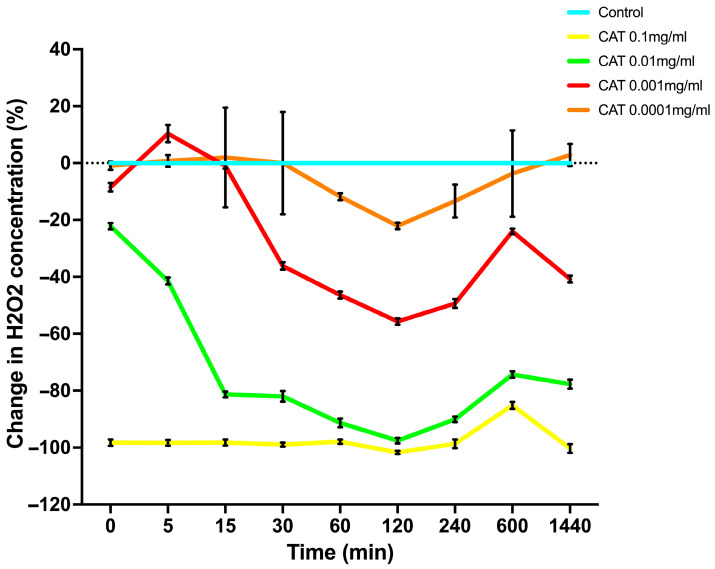
The temporal patterns displaying changes in extracellular H_2_O_2_ concentrations in RAW 264.7 macrophages when treated with catalase. Values represent percentage change in H_2_O_2_ concentration in RAW 264.7 cell culture medium of catalase treated cells, varying in concentration (0.0001, 0.001, 0.01, and 0.1, mg/mL) relative to untreated cells over 1440 min. Results are expressed as mean ± standard deviation from three experiments each measured in triplicate.

The relative capacity of catalase to modulate extracellular H_2_O_2_ levels in RAW 264.7 macrophages over a range of concentrations is shown in Figure 1. The temporal patterns of H_2_O_2_ degradation and the subsequent time-dependent return to near-baseline levels (0% change), characteristic of control RAW 264.7 cells, differed depending on the catalase concentrations used to alter media H_2_O_2_ levels.

The temporal pattern of progressive H_2_O_2_ decline and the magnitude of its reduction depended on the concentration of catalase added to the medium. Specifically, a range of catalase concentrations from 0.0001–0.01 mg/mL produced concentration-dependent reductions of −2.63%, −8.25%, and −22.40%, respectively. In contrast, the highest catalase concentration (0.1 mg/mL) produced almost complete H_2_O_2_ degradation (approximately 95.5%). At the initial time point (referred to as 0 min in Figure 1), catalase at 0.0001 mg/mL produced only a minimal decrease in baseline H_2_O_2_ levels (−2.63%), whereas 0.001 mg/mL and 0.01 mg/mL resulted in larger reductions of −8.25% and −22.40%, respectively. The most substantial decline was observed with 0.1 mg/mL catalase, reaching −95.50% at the initial time point. Notably, a gradual recovery toward basal H_2_O_2_ levels was observed over time, with recovery timing corresponding to the extent of the initial decrease in H_2_O_2_ induced by catalase.

These findings reflect the enzymatic activity of catalase in degrading H_2_O_2_ to water and oxygen through a two-step mechanism involving four heme–iron subunits associated with His75 and Asn148 binding sites [17]. The efficiency of extracellular H_2_O_2_ removal was therefore related to the concentration of catalase added to the cell culture medium. Based on the distinct temporal patterns of H_2_O_2_ degradation followed by partial recovery, three catalase concentrations were selected for subsequent experiments to evaluate their effects on intracellular signaling pathways.

### 3.2. Catalase-Mediated Extracellular H_2_O_2_ Depletion Activates MAPK Signaling in RAW 264.7 Cells

**Figure 2:**
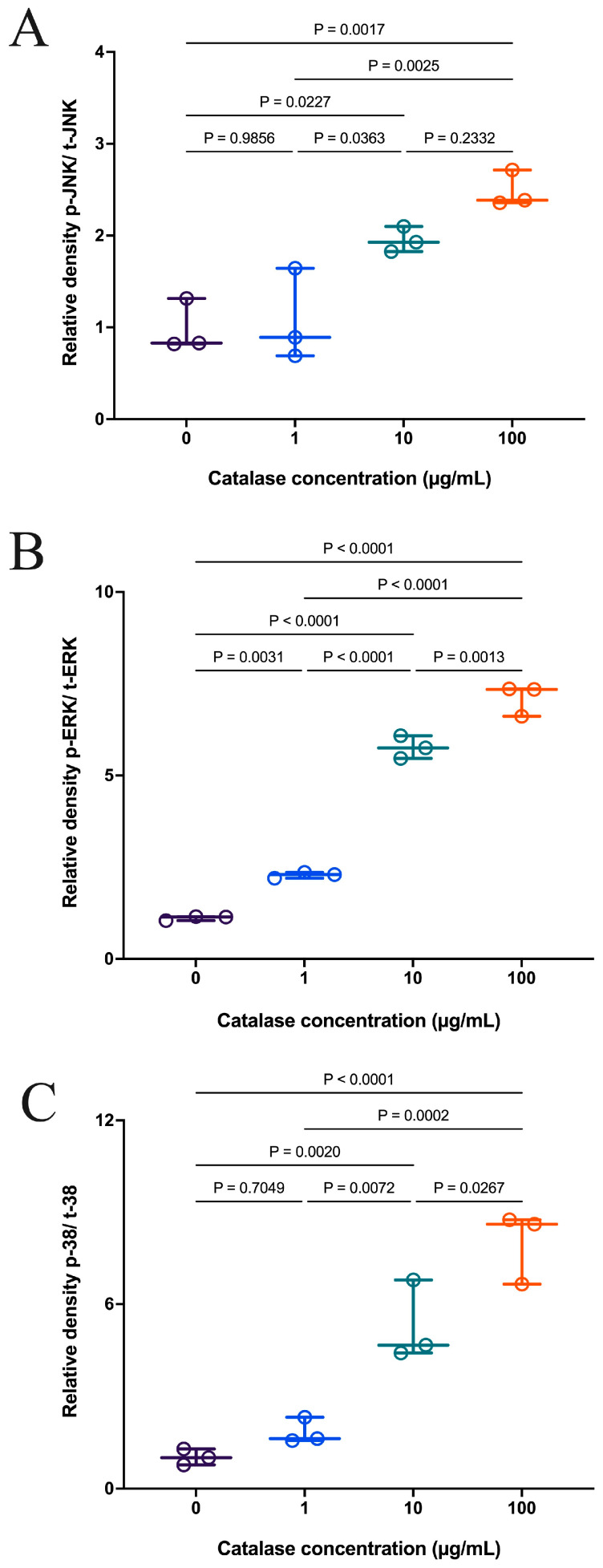
Effect of catalase on (**A**) JNK, (**B**) ERK, and (**C**) p38 signaling in RAW 264.7 cells. Data are presented as individual biological replicates (dots) with mean ± SD (*n* = 3). Statistical significance was determined by one-way ANOVA followed by Tukey’s multiple comparisons test (*p* < 0.05).

The effects of extracellular catalase treatment on MAPK (mitogen-activated protein kinase) signaling activation in RAW 264.7 macrophages are shown in Figure 2. After one hour of treatment with a range of catalase concentrations, the phosphorylation levels of JNK, ERK, and p38 MAPKs were measured. Catalase treatment induced phosphorylation of JNK (Figure 2A), ERK (Figure 2B), and p38 (Figure 2C) in a concentration-dependent manner, although the degree of activation varied among the individual kinases. These results indicate that the extracellular depletion of H_2_O_2_ was sufficient to activate components of the MAPK signaling pathway in macrophages.

### 3.3. Catalase-Mediated Extracellular H_2_O_2_ Depletion Activates NF-κB Signaling in RAW 264.7 Cells

**Figure 3:**
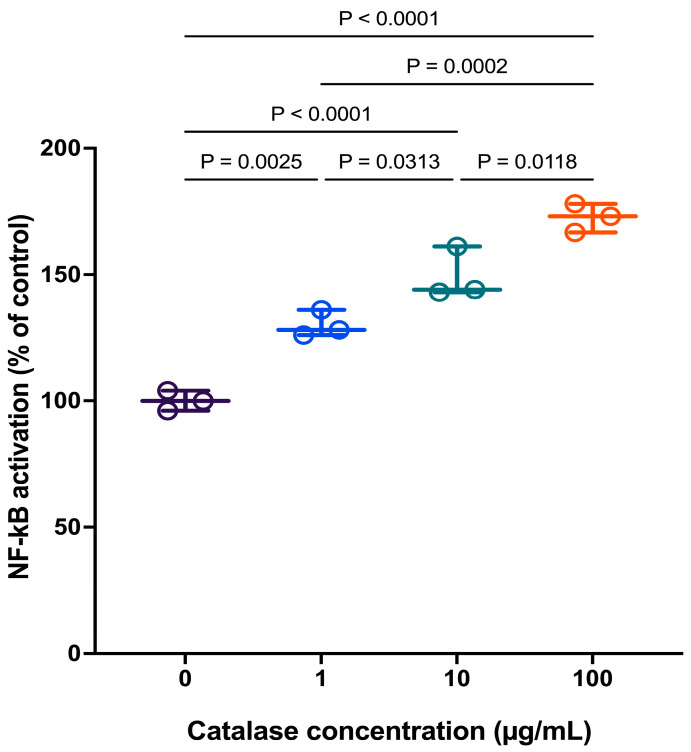
Effect of catalase on p65 nuclear translocation (% of control) in RAW 264.7 cells. Data are presented as individual biological replicates (dots) with mean ± SD (*n* = 3). Statistical significance was determined by one-way ANOVA followed by Tukey’s multiple comparisons test (*p* < 0.05).

Catalase-mediated extracellular H_2_O_2_ depletion was shown to activate p65 nuclear translocation 1.5 h after treatment in RAW 264.7 cells (Figure 3). The results, expressed as a percentage of control, demonstrate that catalase treatment promoted p65 nuclear translocation in a concentration-dependent manner, indicating that activation of the NF-κB signaling pathway occurred relatively early following H_2_O_2_ depletion.

### 3.4. Catalase-Mediated Extracellular H_2_O_2_ Depletion Induces Inflammatory and Oxidative Stress Gene Expression

**Figure 4:**
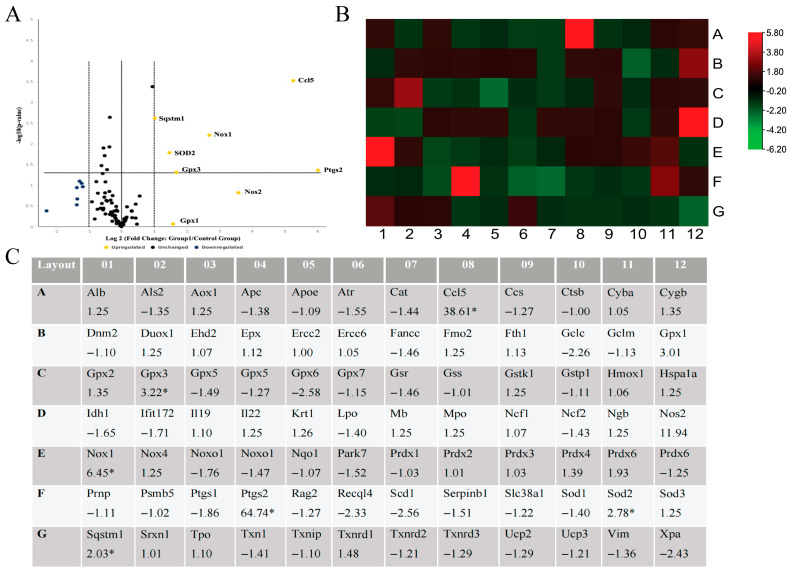
(**A**) The volcano plot identifies significant gene expression changes following treatment with 0.1 mg/mL catalase. The plot displays statistical significance versus fold change on the y- and x-axes, respectively. By combining *p*-value significance with fold regulation, the volcano plot enables the identification of genes that show statistically significant expression changes (*p* < 0.05). (**B**) The heat map visualizes the fold changes in gene expression between the 0.1 mg/mL catalase-treated group and the control for each gene in the array within the context of the array layout. The accompanying table provides the fold regulation data used to construct the heat map, along with comments associated with each gene. (**C**) Table summarizing the fold regulation values corresponding to the heat map and the array layout. Letters on the vertical axis and numbers on the horizontal axis represent the layout of a 96-well plate and do not correspond to experimental variables. * indicates a statistically significant difference (*p* < 0.05). Abbreviation: Alb: Albumin; Als2: ALS2, Rho Guanine Nucleotide Exchange Factor; Aox1: Aldehyde Oxidase 1; Apc: Adenomatous Polyposis Coli; Apoe: Apolipoprotein E; Atr: ATR Serine/Threonine Kinase; Cat: Catalase; Ccl5: C-C Motif Chemokine Ligand 5; Ccs: Copper Chaperone for Superoxide Dismutase; Ctsb: Cathepsin B; Dnm2: Dynamin 2; Duox1: Dual Oxidase 1; Ehd2: EH Domain Containing 2; Epx: Eosinophil Peroxidase; Ercc2: ERCC Excision Repair 2, TFIIH Core Complex Helicase Subunit; Ercc6: ERCC Excision Repair 6, Chromatin Remodeling Factor; Fancc: Fanconi Anemia Complementation Group C; Fmo2: Flavin Containing Monooxygenase 2; Fth1: Ferritin Heavy Chain 1; Gclc: Glutamate-Cysteine Ligase Catalytic Subunit; Gpx2: Glutathione Peroxidase 2; Gpx3: Glutathione Peroxidase 3; Gpx4: Glutathione Peroxidase 4; Gpx5: Glutathione Peroxidase 5; Gpx6: Glutathione Peroxidase 6; Gpx7: Glutathione Peroxidase 7; Gsr: Glutathione-Disulfide Reductase; Gss: Glutathione Synthetase; Gstk1: Glutathione S-Transferase Kappa 1; Gstp1: Glutathione S-Transferase Pi 1; Idh1: Isocitrate Dehydrogenase (NADP^+^) 1; Ift172: Intraflagellar Transport 172; Il19: Interleukin 19; Il22: Interleukin 22; Krt1: Keratin 1; Lpo: Lactoperoxidase; Mb: Myoglobin; Mpo: Myeloperoxidase; Ncf1: Neutrophil Cytosolic Factor 1; Ncf2: Neutrophil Cytosolic Factor 2; Nox1: NADPH Oxidase 1; Nox4: NADPH Oxidase 4; Noxa1: NADPH Oxidase Activator 1; Noxo1: NADPH Oxidase Organizer 1; Nqo1: NAD(P)H Quinone Dehydrogenase 1; Park7: Parkinson Disease Protein 7 (DJ-1); Prdx1: Peroxiredoxin 1; Prdx2: Peroxiredoxin 2; Prdx3: Peroxiredoxin 3; Prdx4: Peroxiredoxin 4; Prnp: Prion Protein; Psmb5: Proteasome Subunit Beta 5; Ptgs1: Prostaglandin-Endoperoxide Synthase 1 (COX-1); Ptgs2: Prostaglandin-Endoperoxide Synthase 2 (COX-2); Rag2: Recombination Activating Gene 2; Recql4: RecQ Like Helicase 4; Scd1: Stearoyl-CoA Desaturase 1; Serpinb1: Serpin Family B Member 1; Slc38a1: Solute Carrier Family 38 Member 1; Sod1: Superoxide Dismutase 1; Sqstm1: Sequestosome 1; Srxn1: Sulfiredoxin 1; Tpo: Thyroid Peroxidase; Txn1: Thioredoxin 1; Txnip: Thioredoxin Interacting Protein; Txnrd1: Thioredoxin Reductase 1; Txnrd2: Thioredoxin Reductase 2; Txnrd3: Thioredoxin Reductase 3; Ucp2: Uncoupling Protein 2; Ucp3: Uncoupling Protein 3. Relative Fold Regulation to Control. Treatment time = 4 h. Experiments were conducted in triplicate.

Real-time PCR demonstrated that depletion of extracellular H_2_O_2_ in RAW 264.7 macrophages induced by catalase (100 µg/mL) at 4 h post-administration resulted in pronounced transcriptional activation of genes involved in inflammatory and oxidative stress responses. Consistent with the volcano plot analysis (Figure 4A), catalase treatment markedly upregulated cyclooxygenase-2 (COX-2/Ptgs2), chemokine ligand 5 (Ccl5), inducible nitric oxide synthase (iNOS/Nos2), and NADPH oxidase (Nox1), with fold increases of 64.74-, 28.61-, 11.94-, and 6.45-fold, respectively.

In parallel, genes associated with antioxidant defence and cellular stress responses, including superoxide dismutase 2 (SOD2), glutathione peroxidases (Gpx1 and Gpx3), and sequestosome-1 (Sqstm1), were upregulated, indicating activation of compensatory antioxidant pathways following H_2_O_2_ depletion. Notably, only a limited number of genes were downregulated, and the majority of transcripts clustered near baseline expression levels, suggesting that catalase-mediated H_2_O_2_ removal elicited a selective, pathway-specific transcriptional response rather than widespread global gene expression changes (Figure 4A,B).

### 3.5. Effects of Removal of H_2_O_2_ by Extracellular Catalase on Nitric Oxide Production in RAW 264.7 Cells

**Figure 5:**
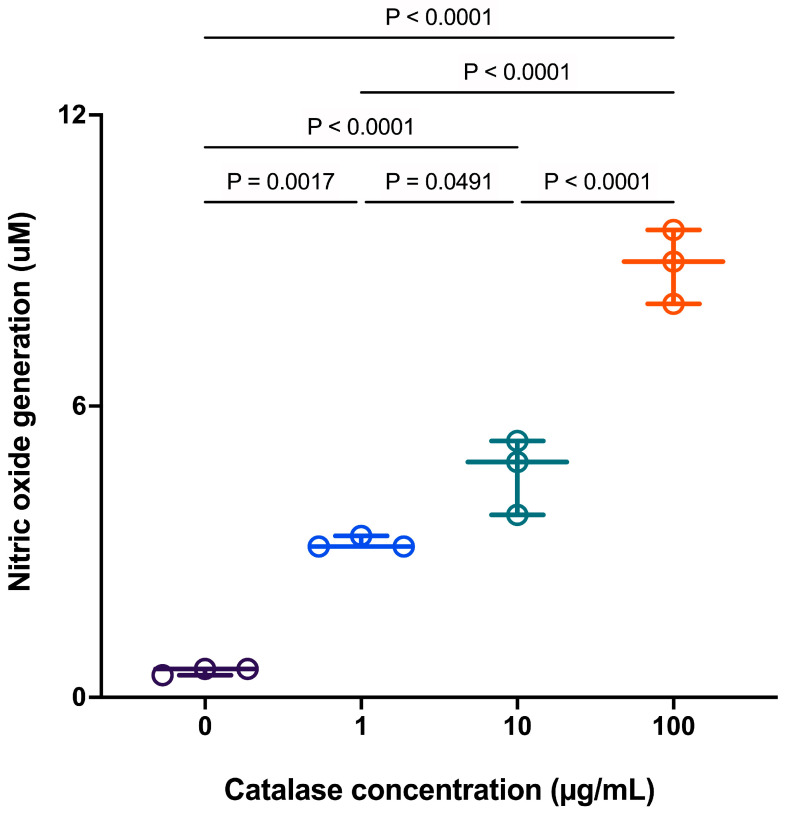
Effect of catalase on nitric oxide (NO) production in RAW 264.7 cells. Data are presented as individual biological replicates (dots) with mean ± SD (*n* = 3). Statistical significance was determined by one-way ANOVA followed by Tukey’s multiple comparisons test (*p* < 0.05).

RAW 264.7 cells cultured in the presence of a range of catalase concentrations for up to 20 h produced a concentration-dependent increase (*p* < 0.05) in nitric oxide (NO) production (Figure 5). This result corresponded with the earlier observation at 4 h of increased inducible nitric oxide synthase (iNOS) gene expression shown in Figure 4B. Pretreatment of cells with catalase to remove extracellular H_2_O_2_ revealed a complex interplay between prolonged depletion of H_2_O_2_ signaling and the regulation of NO production.

**Figure 6:**
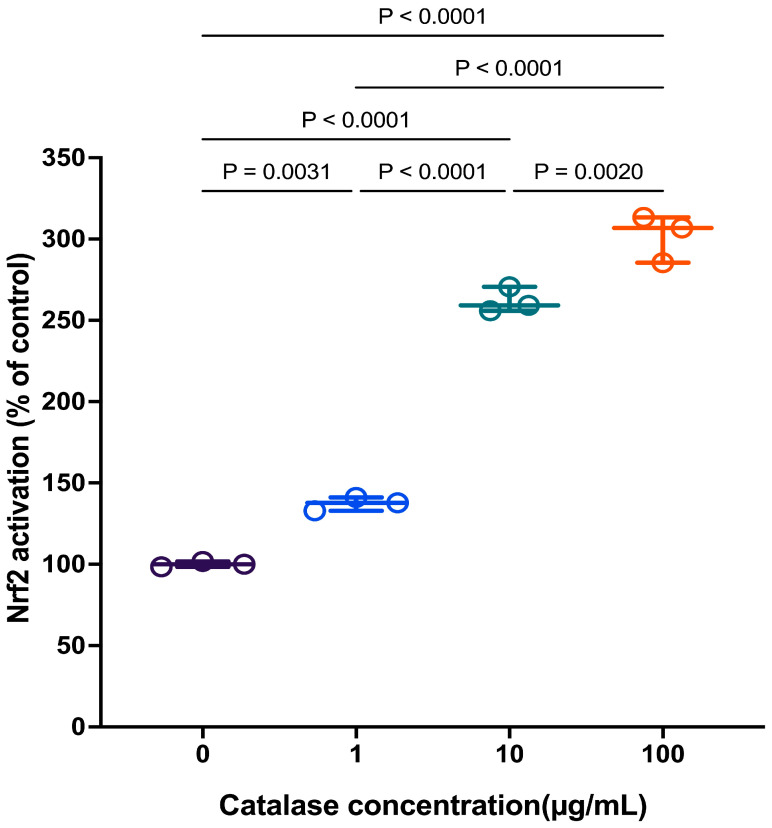
Effect of catalase on Nrf2 translocation (% of control) at t = 24 h in RAW 264.7 cells. Data are presented as individual biological replicates (dots) with mean ± SD (*n* = 3), Statistical significance was determined by one-way ANOVA followed by Tukey’s multiple comparisons test (*p* < 0.05).

The capacity of extracellular catalase to induce Nrf2 nuclear translocation in RAW 264.7 cells after 24 h is presented in Figure 6. Nrf2 levels increased in response to catalase in a concentration-dependent manner. These findings indicate that prolonged catalase treatment, which reduced extracellular H_2_O_2_ levels, was associated with subsequent activation of intracellular redox-sensitive signaling pathways.

**Figure 7:**
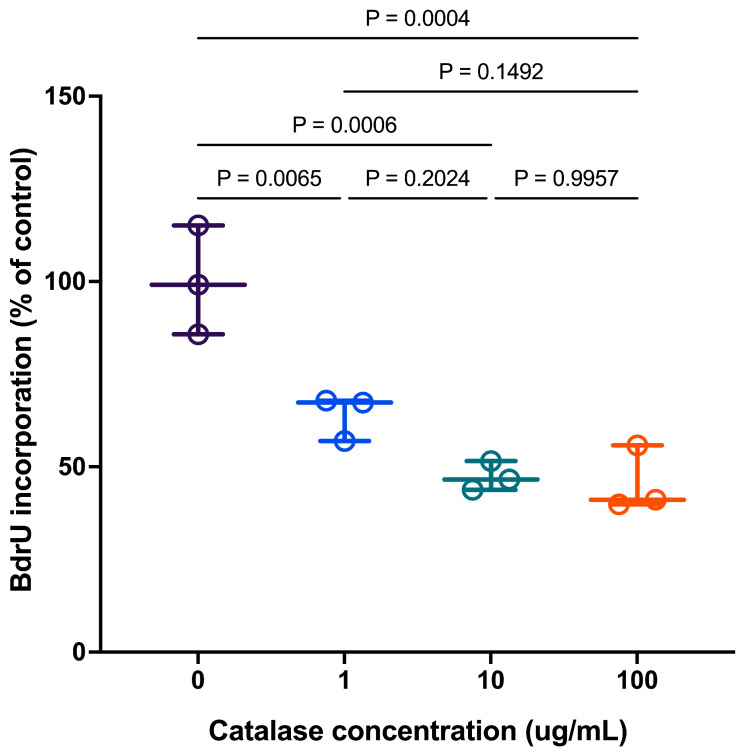
Effect of catalase on RAW 264.7 cell proliferation. Data are presented as individual biological replicates (dots) with mean ± SD (*n* = 3). Statistical significance was determined by one-way ANOVA followed by Tukey’s multiple comparisons test (*p* < 0.05).

Increasing extracellular catalase inhibited macrophage proliferation in RAW 264.7 cells, as assessed by 5-bromo-2′-deoxyuridine (BrdU) incorporation (Figure 7). BrdU incorporation into newly synthesized DNA of replicating macrophages reflects the rate of proliferation during the S phase of the cell cycle. The data show a catalase concentration-dependent decrease in BrdU incorporation, plateauing at 10 µg/mL. At lower concentrations, BrdU incorporation remained relatively high, indicating active cell proliferation. In contrast, higher catalase concentrations significantly (*p* < 0.05) reduced BrdU incorporation, indicating that marked H_2_O_2_ depletion was associated with reduced proliferative capacity. No further change was observed at a 100-fold higher catalase concentration added to the RAW 264.7 cell medium, suggesting that maximal inhibition of cell proliferation had been reached.

RAW 264.7 cell viability remained >90% across all catalase concentrations tested, indicating that the reduction in cell proliferation was not due to cell death. These findings indicate that catalase primarily acts to regulate extracellular H_2_O_2_ levels at the concentrations tested, reducing proliferative activity without inducing cytotoxic effects.

With increasing macrophage exposure to catalase, the removal of H_2_O_2_ below a threshold concentration led to a loss of redox signaling required to promote macrophage proliferation. This inhibited cell growth; however, the results reported here also show that the catalase concentrations and duration of exposure were not sufficient to cause cell death.

## 4. Discussion

Previous studies have proposed that both oxidant and antioxidant signaling are integral components of redox homeostasis [17]. The concept of a “Golden Mean” has been introduced to describe a dynamic equilibrium in which opposing redox forces operate simultaneously, such that deviation toward either excessive oxidation or excessive reduction disrupts cellular homeostasis. Under physiological conditions, transient redox challenges shift this balance, but compensatory feedback mechanisms normally restore equilibrium. However, when a redox perturbation persists or when compensatory responses are inappropriate, sustained alterations in cellular homeostasis may occur, often manifesting as maladaptive inflammatory or stress responses [18].

Hydrogen peroxide (H_2_O_2_) is increasingly recognized as a signaling molecule rather than merely a metabolic byproduct. Similar to nitric oxide, H_2_O_2_ functions as a diffusible redox messenger capable of traversing biological membranes and modulating intracellular signaling pathways in both autocrine and paracrine contexts [19,20]. Immune cells, including macrophages, deliberately generate low levels of H_2_O_2_ during activation and growth factor stimulation, where it serves to fine-tune downstream signaling cascades [21]. Importantly, this extracellular H_2_O_2_ pool contributes to the local redox environment and acts as a tonic homeostatic signal, supporting inflammatory readiness and cellular proliferation under physiological conditions [21,22].

In the present study, we demonstrate that the selective depletion of extracellular H_2_O_2_, achieved through catalase treatment represents a distinct redox perturbation that profoundly alters macrophage signaling. Exposure of RAW 264.7 macrophages to catalase resulted in rapid, concentration- and time-dependent removal of extracellular H_2_O_2_, accompanied by marked changes in intracellular signaling and gene expression. Notably, scavenging extracellular H_2_O_2_ alone, without direct intracellular oxidant exposure, was sufficient to trigger robust activation of MAPK signaling (ERK, JNK, and p38) and the NF-κB pathway, as evidenced by increased phosphorylation and nuclear translocation of the p65 subunit. These findings are consistent with earlier reports describing paradoxical activation of MAPK pathways following exogenous catalase treatment in macrophages [23].

Rather than representing a simple reversal of oxidative stress, these results suggest that macrophages perceive the abrupt loss of extracellular H_2_O_2_ as a stress or danger signal. Under homeostatic conditions, basal extracellular H_2_O_2_ likely maintains a tonic signaling state that constrains inflammatory activation. Its rapid removal disrupts this equilibrium, initiating compensatory intracellular signaling responses. Consistent with this interpretation, activation of MAPK and NF-κB pathways occurred in a temporally overlapping manner, supporting the view that these pathways act as parallel responses to extracellular redox imbalance, rather than as components of a strictly linear signaling hierarchy.

Activation of NF-κB and MAPK pathways following extracellular H_2_O_2_ depletion was accompanied by pronounced transcriptional upregulation of inflammatory and oxidative stress–related genes, including inducible nitric oxide synthase (iNOS) and cyclooxygenase-2 (COX-2), both canonical NF-κB target genes. These observations align with previous studies demonstrating that catalase-induced iNOS and COX-2 expression in macrophages is NF-κB dependent, and that pharmacological inhibition of NF-κB abrogates this response [23]. As a functional consequence of iNOS induction, catalase-treated macrophages exhibited increased nitric oxide (NO) production. Together, these findings reinforce the concept that both excessive and insufficient ROS availability can disrupt redox homeostasis, eliciting inflammatory signaling as a compensatory response.

In parallel with inflammatory activation, catalase-mediated extracellular H_2_O_2_ depletion also promoted activation of nuclear factor erythroid 2–related factor 2 (Nrf2), a master regulator of antioxidant defense. While the present study does not establish a direct causal mechanism, a plausible explanation is that increased NO production contributes to Nrf2 activation, potentially through redox-sensitive modification of Keap1 or related regulatory proteins [24]. Importantly, Nrf2 activation occurred alongside NF-κB and MAPK signaling, consistent with a model in which extracellular redox perturbation initiates coordinated inflammatory and antioxidant programs aimed at restoring cellular equilibrium and limiting self-inflicted oxidative damage [25].

A notable biological outcome of extracellular H_2_O_2_ depletion was the suppression of macrophage proliferation. Catalase-treated RAW 264.7 cells exhibited reduced DNA synthesis and impaired proliferative capacity, supporting the notion that basal extracellular H_2_O_2_ contributes to cell cycle progression in macrophages [21,22]. This observation is consistent with a broader literature demonstrating that low-level ROS function as mitogenic signals across diverse cell types, whereas their removal can lead to growth arrest or apoptosis [21]. Similar effects have been reported in vascular smooth muscle cells, where endogenous H_2_O_2_ is required for proliferation and its scavenging suppresses growth [21].

While reduced proliferation may reflect, in part, direct effects of extracellular redox perturbation on growth-related signaling, our data also support a role for secondary autocrine mechanisms. Catalase-induced upregulation of COX-2 and iNOS suggests increased production of inflammatory mediators such as prostaglandin E_2_ and NO, which are known to exert anti-proliferative effects in macrophages and other cell types. Thus, inhibition of proliferation likely reflects the combined effects of altered extracellular redox signaling and downstream inflammatory activation, rather than a single dominant pathway.

Taken together, our findings support a model in which extracellular H_2_O_2_ functions as a physiological redox signal essential for macrophage homeostasis. Its depletion initiates a coordinated intracellular response involving MAPK, NF-κB, NO, and Nrf2 signaling, ultimately reshaping inflammatory, antioxidant, and proliferative programs. This framework emphasizes the importance of extracellular redox balance in immune regulation and provides a mechanistic foundation for understanding how changes in the oxidative environment influence macrophage function under physiological and pathological conditions.

## 5. Conclusions

In conclusion, our findings demonstrate that extracellular H_2_O_2_ plays an important physiological role in regulating intracellular signaling pathways in macrophages. Under normal conditions, basal H_2_O_2_ helps maintain redox homeostasis by modulating key signaling pathways such as MAPKs and NF-κB. Catalase-mediated depletion of extracellular H_2_O_2_ disrupted this balance and triggered a coordinated cellular response, including activation of pro-inflammatory transcription factors, upregulation of inflammatory genes (iNOS and COX-2), increased nitric oxide production, and Nrf2-mediated stress adaptation. The time-dependent effects on multiple signaling pathways observed in this study highlight the importance of extracellular redox signals in coordinating immune responses. Furthermore, these findings suggest that changes in extracellular reactive oxygen species levels can significantly influence immune cell behavior and redox-dependent regulation.

## Data Availability

The data are available from the corresponding author, David. D. Kitts. (david.kitts@ubc.ca), upon request.

## Supplementary Materials

The following supporting information can be downloaded at: https://www.mdpi.com/article/10.3390/antiox15030366/s1, Table S1. Fold regulation comparison and *p*-value for Real time PCR results.
