# Supplementary material for: Analysis of Catalase-Induced Activation of Intracellular Cell Signaling in Macrophages
Source: Antioxidants (Basel). 2026 Mar 13;15(3):366. doi: 10.3390/antiox15030366 (PMC13023523; doi:10.3390/antiox15030366)
Supplement: Supplementary file 1 [file antioxidants-15-00366-s001.zip › antioxidants-4187229-supplementary.pdf]

Supplementary Table S1. Fold regulation comparison and p-value for Real time PCR results

Gene Symbol	Fold Regulation	p-Value
Ccl5	38.61	0.000300
Gpx3	3.22	0.049263
Nox1	6.45	0.006200
Ptgs2	64.74	0.043793
Sod2	2.78	0.016429
Sqstm1	2.03	0.002432

Fold-Change (2^ (- Delta Delta CT)) is the normalized gene expression (2^(- Delta CT)) in the Test Sample divided the normalized gene expression (2^ (- Delta CT)) in the Control Sample. Fold-Regulation represents fold-change results in a biologically meaningful way. Fold-change values greater than one indicates a positive- or an up-regulation, and the fold-regulation is equal to the fold-change. Fold-change values less than one indicate a negative or down-regulation, and the fold-regulation is the negative inverse of the fold-change.

The p values are calculated based on a Student's t-test of the replicate 2^ (- Delta CT) values for each gene in the control group and treatment groups.
